# Watson’s Practice of Physic

**Published:** 1872-08

**Authors:** 


### Books Reviewed.

*The Principles and Practice of Physic.* By Sir THOMAS WATSON, Bart. M. D., F. R. S. In two volumes, edited with additions and numerous illustrations, from the Fifth English Edition, by HENRY HARTSHORNE, A. M., M. D. Philadelphia: HENRY C. LEA, 1872. Buffalo: T. BUTLER & SON.

The years which have elapsed since Watson's Practice of Physic was first placed in the hands of the profession, has witnessed many changes in the art of dealing with disease. Much that was in former years deemed essential, is now looked upon with that curiosity with which we view some relic of the days of barbarism.

We wish all of our readers could have the pleasure of makihg a comparison between the editions of 1844 and of 1872. We assure them that it would afford them food for profitable reflection.

The present American Edition is edited by a physician, whose experience as a writer, practitioner, and teacher has made him in every way fitted for the duty. The work is amply illustrated, the illustrations adding greatly to its value especially as a text-book for the student, or book of reference for the young practitioner. The pathology of disease was written by Watson, in the very first edition of his work with unsurpassed accuracy and beauty; it was our Bible of Medicine when a student, and though we early learned not to follow his teaching in the treatment of disease, still we have never ceased to admire his plain and attractive descriptions of its causes and nature. That a large amount of additional matter has been incorporated in the work is shown by the addition of the second volume of the full size of the first issue. Many diseases not previously mentioned are now fully discussed, and valuable additions have been made to the lectures as they formerly appeared, so that it is complete in scope and extent. This book as it is now offered to the profession, is vastly more valuable than before; it contains all the excellencies of former editions, and perhaps none of the faults. It is brought down to our time and country, and is a safe guide in the *treatment* of disease, a statement which we do not think could truthfully be made of the early issues. This was the misfortune of the day, and not the fault of the author. Bleeding, blistering, antimony and calomel, were the staples in the treatment of nearly all diseases, nature had rarely been allowed to show the power of her influence.

Time has changed the general teaching and added what was lacking, so that Watson's Practice of Physic, by Hartshorne is perhaps in all respects the most complete and comprehensive work of the kind in this language, and as well adapted to the wants of physicians and students, as can be in the present state of knowledge.

